# Coping with Caregiving Stress in Families of Children with Congenital Heart Disease: A Qualitative Study

**DOI:** 10.30476/IJCBNM.2020.83029.1113

**Published:** 2020-04

**Authors:** Zahra Dalir, Abbas Heydari, Hossein Kareshki, Zahra Sadat Manzari

**Affiliations:** 1 Nursing and Midwifery Care Research Center, Mashhad University of Medical Sciences, Mashhad, Iran; 2 Department of Counseling and Educational Psychology, School of Educational Sciences and Psychology, Ferdowsi University of Mashhad, Mashhad, Iran

**Keywords:** Child, Congenital heart defects, Coping, Family, Stress

## Abstract

**Background:**

The families of children with congenital heart disease experience significant stress as to the care of the child and need to cope with stress. Accordingly, understanding of how families cope and use coping strategies is more important to help them better cope with stressful situations caused by caregiving. This study aimed to explore coping strategies used by families in the face of caregiving stress.

**Methods:**

This qualitative study was conducted on 40 eligible participants from the families of children with congenital heart disease. They were recruited through a purposive sampling method from those referred to hospitals in Mashhad, Iran. In-depth and semi-structured interviews were used for data collection from November 2017 to December 2018. The data were analyzed using conventional content analysis, and MAXQDA software (Ver.2010) was used to manage the data encoding process.

**Results:**

According to the results, effort to maintain well-being emerged as the main theme which included five categories: “spirituality in caregiving”, “acceptance and adjustment”, “optimism and hopefulness”, “self-control and patience”, and “ management of psychological needs”.

**Conclusion:**

Families used various coping strategies including spirituality, acceptance, optimism, patience, and management of psychological needs based on their beliefs, attitudes, abilities, and available resources for coping with caregiving stress. The results can help the nurses and health care professionals to develop appropriate educational, supportive, and psychological interventions based on the family’s needs to cope effectively with caregiving stress.

## INTRODUCTION

Congenital heart disease (CHD) is a common congenital defect with geographic variations. The birth prevalence rate of this disease in North America, Europe, and Asia is reported at 6.9, 8.2, and 9.3 per 1000 live birth, respectively. ^1^ In Iran, the prevalence rate is less than 10 per 1000 live birth (less than 1%). ^2^ Children with CHD may require several times of hospitalization, interventional therapies, surgeries, and follow-ups during their life. ^3^ Advanced medical and surgical practices have led many congenital heart defects to be managed as a chronic disease, while they were considered as life-threatening previously. ^4^ However, the child’s chronic illness can cause challenges above usual parental responsibilities due to managing the child’s illness-specific needs, integrating the needs into family life, social constraints, and influencing psychological health of parents. ^5^

The diagnosis of CHD has considerable psychological effects on the parents. ^6^ Accordingly, 25-50% of the parents of children with severe CHD reported clinical symptoms of anxiety or depression. ^7^ Regardless of the type of CHD and the child’s age, families experience significant stress in child caregiving, ^6^ which imposes an emotional, financial and family member burden. Moreover, the presence of a child with CHD in the family may lead other siblings to get stressed. ^4^ Therefore, it might affect the whole family. ^8^

Caregiving tasks and responsibilities are stressful for caregivers, and the degree of the impact of stress on the caregivers’ life depends on their coping strategies. ^9^ Systematic review studies on families of children with CHD reported that the family psychosocial coping varies over time, and a successful adaptation to the child’s illness depends on the cohesiveness of the family and their adaptive coping strategies. ^10 , 11^ However, these families are diverse by their needs, experiences, amount of support received and ways of coping. ^10 , 11^ Some quantitative and qualitative studies were conducted on the coping strategies of families/parents of children with CHD. The results showed that these families used emotion-focused or problem-focused coping strategies, such as avoidance and denial, spirituality, acceptance, maintenance of family integrity, and psychological stability in dealing with stress. ^12 - 16^

Moreover, some studies revealed variations in mothers’ and fathers’ coping styles. They used adaptive or maladaptive coping strategies. ^16 , 17^ In general, receiving social support was effective in parental coping. ^12 , 15^ Moreover, support could be achieved through religious beliefs, faith and spirituality. ^10^ In a study, the parents of children with CHD used positive coping strategies for dealing with the challenges of their child’s illness; however, they required coping-focused support. ^16^

Effective coping strategies may reduce distress and improve the ability to tolerate the burden of the disease for parents without affecting them by psychiatric illnesses, ^18^ and may be associated positively with their psychological quality of life. ^19^ However, cultural beliefs may influence on the appraising of stress, receiving social support and using coping strategies by caregivers. ^20^

Although there are studies about coping in families of children with CHD, ^12 - 16^ to the best of our knowledge, there is still a paucity of qualitative research on how these families cope with caregiving stress. ^13 , 16^ Given the prevalence of this disease in Iran, it imposes an emotional and economic burden on the family, health system, and society. Moreover, contexts differ in culture, beliefs, religion, and supportive health care systems, which influence the experiences of families towards stress and coping. ^10 , 11 , 20^ Therefore, it is essential to have a deep and comprehensive understanding of how family cope and use coping strategies in the face of caregiving stress to help them cope better with stressful situations. In this regard, qualitative studies are needed to gain deep insight into the experiences of the families. This study aimed to explore coping strategies used by families of children with CHD in the face of caregiving stress. 

## MATERIALS AND METHODS

This qualitative study was conducted based on a conventional content analysis approach for gaining a richer understanding of the phenomenon and direct information from the participants without imposing theoretical perspectives or preconceived categories, which generates knowledge based on their unique perspectives. ^21^ This study was conducted in Imam Reza and Ghaem hospitals affiliated to Mashhad University of Medical Sciences, and Javad Al-Aeme Cardiovascular Hospital in Mashhad, Iran. Imam Reza Hospital in Mashhad is a center in the northeast of Iran due to having a pediatric congenital heart disease department, so children with CHD from other neighboring cities and provinces are referred for treatment. Participants were families of children with CHD who were selected through purposive sampling with maximum diversity in their educational level and socioeconomic status, as well as child’s age and type of CHD.

The inclusion criteria were Persian-speaking Iranian families who were taking care of their child with CHD, willingness to participate in the study, no confirmed mental disorders in participants, the child’ age range between 6 months to 11 years, and diagnosis of the child’s CHD at least 6 months before our study. 

In-depth and semi-structured interviews were used for data collection from 40 participants, including 27 mothers, 8 fathers, 3 siblings, and 2 grandmothers from November 2017 to December 2018. The interviews were performed individually and recorded by the first author at the appropriate time and a private room in pediatric congenital heart wards in the hospital. 

The interview started with warm up questions, such as *“When/how was your child’s heart disease diagnosed?”* and moved to specific interview questions, such as *“What challenges and stressful situations did/do you face with during taking care of your child?”*, *“How did/do you cope with these difficult and stressful situations?”*, *“What did/do you do to overcome these challenges?”*, *“What did/do you do to get calm and relieved?”*. Depending on the participant’s response, some questions were also asked for clarification and getting more information to resolve the ambiguities, such as *“Can you explain more in details?”*, *“What do you mean by..?”*.

Data collection continued until data saturation and the completion of the codes and categories and no new codes were obtained from the data. Overall, 40 interviews (39 in-person, one telephone) were conducted which lasted between 30-80 mins. It should be noted that there was not any possibility for in-person interview with a child’s sister due to the long distance of the families’ place of residence; therefore, one telephone interview was conducted with her after interviewing the parents. The telephone interview was performed by the first author in 30 min. In this study, 3 participants (siblings) were in the age range of 11-15 years old, and the interview questions were similar to the above-mentioned questions, but they were modified slightly based on their age and understanding: *“What problems and stressful situations did/do you face with because of your sister’s/brother’s disease?”*, *“How did/do you cope with /deal with/overcome the problem and situation?”*. The recorded interviews were transcribed in the shortest possible time, and all the transcripts were imported into MAXQDA software (version 2010, VERBI Software GmbH, Germany) for analysis. 

Graneheim and Lundman’s (2004) qualitative content analysis method with the conventional approach was used to analyze the data. ^22^ The data were collected and analyzed simultaneously. Each interview transcript was read several times to gain a deep understanding of it. Subsequently, the texts related to the participants’ experiences of coping with caregiving stress and using coping strategies were placed in a text to form the analysis unit. Following that, the meaning units were identified and then condensed meaning units and codes were extracted. Similar codes were placed in the subcategories and then similar subcategories were placed in the category. Then, the theme formed from the categories according to latent content and underlying meaning. Eventually, 182 codes, 13 subcategories, 5 categories and a main theme were obtained from data analysis (Table 1).

**Table 1:** Examples of the meaning units, condensed meaning units, codes, and subcategories

Meaning unit	Condensed meaning unit	Code	Subcategory	Category
It took me time to accept that I had to raise my child with such conditions. Now, I have to compromise on his conditions	Accepting to raise a sick child and compromising on his conditions over time	Accepting the child’s illness Compromising on the child’s conditions	Accepting the child’s conditions	Acceptance and adjustment
We don’t attend any party for prevention of my child cold; it was very hard at first, but we got used to, and entertain ourselves with other things	Getting used to the limitations of living with a sick child, and changing entertainments	Habituating with the limitations of life Modifying life conditions	Adjusting to the life conditions	

In order to ensure the trustworthiness and rigor of the qualitative data, we used Lincoln and Guba’s (1985) criteria including credibility, dependability, confirmability, and transferability in this study. ^23^ Data credibility was obtained through prolonged engagement with the participants and data, member checking by participants’ feedback to the interview text, and sampling with maximum diversity in education and socioeconomic status of participants and the child’s CHD. Dependability was achieved by an external reviewer doing peer-review, so that the texts of some interviews, along with codes, categories, and emerged themes were reviewed and confirmed by three experts in qualitative research who were not on the research team. The confirmability was achieved by audit trial and the transferability of the results was obtained using full description of the context, the process of sampling participants, as well as data collection and analysis.

This study was approved by the Ethics Committee of Mashhad University of Medical Sciences, Mashhad, Iran (IR.MUMS.REC.1396.256). Written informed consent was obtained from all the participants before the interview, and they were informed about how their confidentiality of the data would be preserved. Moreover, they were assured of the right of withdrawal from the study at any time. It should be noted that for participants aged 11-15 years old, written informed consent was obtained from their parents.

## RESULTS

This study included 40 participants (27 mothers, 8 fathers, 3 siblings and 2 grandmothers). Table 2 displays the demographic characteristics of the participants. According to data analysis, 13 subcategories and 5 categories and a main theme entitled ‘ effort to maintain well-being’ were emerged. Five categories included spirituality in caregiving, acceptance and adjustment, optimism and hopefulness, self-control and patience, and management of psychological needs (Table 3).

**Table 2:** Demographic characteristics of the participants

Description	Mothers	Fathers	Siblings	Grandmothers
	Mean±SD	Mean±SD	Mean±SD	Mean±SD
Age				
Range(years)	(20-45)	(32-42)	(11-15)	(56-60)
	32.48±6.47	37.88±4.64	12.67±2.08	58±2.82
	**N (%)**	**N (%)**	**N (%)**	**N (%)**
Education level				
Elementary	3 (11.11)	1 (12.50)	2 (66.70)	2 (100)
High school	13 (48.15)	3 (37.50)	1 (33.30)	
University	11 (40.74)	4 (50)		
Job				
Housewife	16 (59.26)			2 (100)
Employer	9 (33.33)	4 (50)		
Worker/free job	2 (7.41)	4 (50)		
Student			3 (100)	
Residential place				
Mashhad	10 (37.10)	4 (50)	2 (66.70)	2 (100)
Other cities	17 (62.90)	4 (50)	1 (33.30)	

**Table 3:** Main subcategories, categories, theme, and emerged from data analysis

Subcategories	Categories	Theme
Asking God for help	Spirituality in caregiving	Effort to maintain well-being
Trusting in God’s will		
Relief caused by spirituality		
Accepting of the child’s conditions	Acceptance and adjustment	
Adjusting to the life condition		
Optimistic attitude towards care and life	Optimism and hopefulness	
Hopeful about future		
Comforting as a source of hope		
Trying to be self-controlled in caregiving	Self-control and patience	
Being patient in hard conditions of caregiving		
Seeking support	Management of psychological needs	
Releasing emotions		
Ability to meet one’s own and family members’ psychological needs		

### *1. Spirituality in Caregiving*

This category includes three subcategories namely asking God for help, trusting in God’s will, and relief caused by spirituality; it was indicated that religious and spiritual beliefs deeply affect the families’ life, and these beliefs are more highlighted in stressful situations.

*1.a. Asking God for Help*

Almost all participants asked God for help in difficult situations of caregiving and vowed for their child’s cure and health. 

*“… I don’t know what to do when I see my child is in trouble. I can not do anything about it, so I ask God for help in these situations and even make a vow” (P5, father, 37 y/o)*.

*1.b. Trusting in God's Will*

The majority of the participants stated that their child’s illness was God’s will, and they accepted this God-given destiny. Moreover, they were hopeful for child’s healing by God’s help. 

*“…When my child was getting ready to go into the operating room, I asked God: why don’t you show me your miracle? My husband said: this is a miracle that our child can now walk; we cannot do anything because it is God’s will, and whatever God does we have to accept” (P7, mother, 32 y/o)*.

*“… God is great; I comfort myself with these hopeful self-talks. This child is a gift from God; God himself protects my child. If God wishes, my child may live for several years with a single ventricle in the heart, like a person with an eye” (P12, mother, 37 y/o)*. 

Moreover, some participants believed that in difficult and stressful situations, they should trust in God and ask him for patience:

*“…When the child is in trouble, we must have trust in God and be strong and faithful to have the power to continue” (P18, father, 42 y/o)*.

*“…Sometimes, I can’t take care of my child well; we have to ask God for patience and I believe that God gives the patience to me” (P17, mother, 36 y/o)*.

*1.c. Relief Caused by Spirituality*

The majority of the participants believed that praying and reciting Holy Quran, especially in stressful situations of caregiving, are relaxing and energetic.

*“…When my child is in trouble, I have a lot of stress, so I pray; then, my child and I will get so calm. I feel this calmness perfectly” (P20, mother, 34 y/o)*.

*“…When I am so bored of caregiving, I recite Holy Quran, that makes me feel calm and relaxed and gives me energy to take care of my baby” (P1, mother, 37 y/o)*.

### *2. Acceptance and Adjustment*

One of the coping strategies used by the participants was the acceptance of and adjustment to difficult situations. This category included two subcategories of accepting of the child’s conditions and adjusting to the life conditions which indicate the gradual acceptance and adjustment of participants to the stressful conditions of caregiving.

2.a. Accepting of the Child’s Conditions

It can be assumed that after the denial and anger stages, the participants accepted the reality, the child’s illness, and his/her condition.

*“…When I found out that my baby had a heart problem, I was very shocked; I could not accept it at all. It was very hard. It took me a long time to cope with this problem. I accepted that I had to raise him with such conditions, and now I have to compromise on my child’s conditions” (P17, mother, 36 y/o)*.

2.b. Adjusting to the Life Conditions

Some participants stated that, despite having a sick child in the family, they gradually habituated with the changes and limitations in their lives caused by the child’s illness, and they adjusted themselves to these limitations. 

*“…We have to stay at home in the winter; otherwise, my child will catch a cold. We don’t attend any family reunions and party; it was very hard at first, but then we got used to do it. We entertain ourselves at home by watching TV” (P1, mother, 37 y/o)*.

*“…I asked my husband to move our house closer to the hospital; then, I could take care of my other son too. However, it was not possible. We must adjust ourselves to our child’s illness and life conditions” (P30, mother, 33 y/o)*.

### *3. Optimism and Hopefulness*

Optimism and hope are other strategies that families used in the face of their problems in life and child’s caregiving. This category included three subcategories, namely optimistic attitudes towards care and life, hopeful about future, and comforting as a source for hope. Having an optimistic attitude reduced the impact of negative thoughts and made the participants hopeful.

3.a. Optimistic Attitude Towards Care and Life

Optimism involved having an optimistic attitude toward the improvement of the child and life conditions which is achieved by positive thoughts and emphasizing the positive aspects of life. Some participants were optimistic about their child’s recovery in the future by comparing their child’s condition with other children with even worse conditions.

*“…I’m not too worried. I feel my child’s condition is better than many other children; my child is going to undergo surgery to get better. I’m praying, so that my baby will be cured and I’m sure that my child will stay alive after surgery” (P32, mother, 20 y/o)*.

Some participants considered the positive aspects of their child’s disease and mentioned their positive attitude on life as well as the changes occurred in their life.

*“…Sometimes, I think that my child’s disease made me and my husband get closer, and he pays more attention to our life. If it wasn’t for the sake of the child, we were stuck in a routine repetitive relationships with no love” (P23, mother, 31 y/o)*.

3.b. Hopeful About the Future

The child’s gradual recovery and comparison of the child’s condition with other children made the family hopeful about the future and tried to continue the treatment of the child. 

*“…When I see other children with heart disease who are now grown up, I become hopeful and I feel that my child can be the same, too; I should do whatever I can for my child” (P29, mother, 32 y/o)*.

*“…Now, I give hope to myself that everything will be OK because my sister is now much better than before” (P3, sister, 12 y/o)*.

3.c. Comforting as a Source of Hope

The majority of participants stated the important role of comforting from the spouse, family members, and the physician to make them hopeful and encouraged. 

*“…When I’m tired of caregiving, my husband talks to me more and tries to comfort me by his words; thus, he makes me more hopeful” (P1, mother, 37 y/o)*.

*“…Once, Doctor A. showed me a child and said: look at that child he had the same disease as your son’s illness, but he’s grown up now, so don’t be upset, God is great and we doctors will help you. Doctor’s words made me hopeful” (P15, mother, 33 y/o)*.

### *4. Self-Control and Patience*

This category includes two subcategories, namely trying to be self-controlled in caregiving and being patient in hard conditions of caregiving. Most families tried to be patient and self-controlled to deal with problems caused by caregiving and child’s disease.

4.a. Trying to be Self-Controlled in Caregiving

Some participants tried to control their behavior in difficult situations of caregiving to keep peace in the family and avoid tension in their family members:

*“…I get very sensitive when I am tired and nervous about childcare; I try to control myself and not to beat my children because I will regret later. I don’t want to put my children under tension” (P12, mother, 37 y/o)*.

4.b. Being Patient in Hard Conditions of Caregiving

The majority of the participants believed that they should be patient in taking care of their children and tolerate difficult conditions of caregiving.

*“…Thanks God, I am patient. I believe I have to be patient; sometimes, it may be necessary to stay in hospital for one or two months. I have to tolerate, so that I can take care of my child” (P26, mother, 27 y/o)*.

### *5. Management of Psychological Needs*

Since these families experience a lot of tension and psychological stress in the caregiving process, they have to manage their psychological needs in order to get calm and satisfy their needs. This category includes three sub-categories, namely seeking support, releasing emotions, and having ability to meet one’s own and family’s psychological needs in the caregiving process which reflect the efforts of families to manage their needs.

5.a. Seeking Support

Most participants who were faced with caregiving tension and challenges sought emotional support from the family, physicians, relatives, and friends which would lead to the peace of mind and encouragement.

*“… My mom supports me a lot. Whenever I’m upset, I call my mom. When I call her, she makes me relaxed and calm by affecting my mood” (P6, mother, 23 y/o)*.

*“…Dr. A is very emotional; he talks very carefully and calmly. He reassures and supports me emotionally. That’s why I’ve continued to follow my child’s treatment based on his prescriptions” (P1, mother, 37 y/o)*.

5.b. Releasing Emotions

When some participants faced with stressful situations in the caregiving process, they used adaptive and maladaptive coping strategies to release their emotions and get relaxed. The most adaptive strategies included keeping oneself busy, spending time out of the house, and taking a shower.

*“…When my child’s condition gets worse, I get nervous and watch TV to relax, spend time out of home, or read books” (P2, father, 42 y/o)*.

*“…When I’m nervous and upset, I don’t let my child feels it because he will get upset and nervous, too. I take a shower or chew gum to get calm” (P10, mother, 41 y/o)*.

Some participants used maladaptive coping strategies to release their emotions, such as crying, yelling, and showing aggressive behaviors. 

*“…When I get tired of caregiving, I cry or scream, and shout at my children; in this way, I release myself “ (P15, mother, 33 y/o)*.

5.c. Ability to Meet One’s Own and Family Members’ Psychological Needs

Some participants tried to meet their psychological needs using various ways to relax their mind. These strategies included consuming sedative medications, consulting with psychiatrists, and socializing with friends.

*“…I’m very nervous because of my child’s illness. Sometimes, I take sedative pills that psychiatrist has prescribed. When I get better, I stop taking them” (P24, mother, 42 y/o)*.

*“…I don’t like to do something when I am confused. I try to refresh my mood by going to the parties and socializing with friends; it makes me feel good” (P4, mother, 32 y/o)*.

Some participants took actions to meet the needs of their other children and pay more attention to them.

*“…Since I got engaged too much in taking care of my child, I paid less attention to my eldest son, and he got sick. From then on, I try to pay more attention to him, play with him, read bedtime stories, and hug him more” (P23, mother, 31 y/o)*.

## DISCUSSION

According to the results of this study, the families of children with CHD used various coping strategies to cope with caregiving stress. These strategies are classified into five categories, namely spirituality in caregiving, acceptance and adjustment to conditions, optimism and hopefulness, self-control and patience, and management of psychological needs. 

In the present study, spirituality played an important role when families faced stressful situations in caregiving. Spiritual beliefs and religious practices were effective in creating relief, calmness and giving energy, patience, as well as hope for the child’s recovery. It can be said that the religious culture of Iranian society has caused spirituality to be seen in their speech, behavior, and beliefs. Moreover, they use it as an important strategy in coping with stress. The results of studies in other cultures also showed that believing in God, God’s will, and His help as well as praying were important factors for coping with stress in parents of children with heart diseases and chronic diseases. ^24 , 25^ The role of spirituality was remarkable in providing emotional support and calmness. ^25 , 26^ In a study in Thailand, traditional belief of parents in supernatural or sacred powers was effective to get relief and comfort. ^27^ In a study in Hong Kong, fathers of children with cancer performed religious ceremonies like incense burning and offered gifts to their ancestors after confirming the child’s diagnosis. These ceremonies made them hopeful about their child’s recovery. ^28^ In a study, mothers raised their patience through prayer and attending the religious ceremonies. Applying religious teachings and believing in patience were effective strategies in providing optimal care. ^26^ In a study on Iranian Muslim patients with chronic disease, spirituality meant relationship with God, trust in God, spiritual satisfaction, and strong religious beliefs as believing in God’s mercy and omnipotence. ^29^ The above-mentioned points indicate the importance of spirituality and religious beliefs as coping strategy in the face of caregiving stress in different cultures and religions. Although religious practices and ceremonies are more commonly used in non-western cultures, ^30^ it is important to consider the cultural and religious context of individuals in their ability to cope with stress.

Participants in this study needed time to accept their child’s disease and adjust to limitations caused by the child’s illness in their life. In a study, some mothers tended to accept their child’s condition in the process of adaptation to their child’s heart disease. ^31^ The results of a study revealed that mothers also accepted the limitations and new conditions over time after experiencing emotional ups and downs. ^26^ In a study, Thai families of children with CHD believed that their child’s heart disease was caused by *“Karma”* based on their Buddhist beliefs. Therefore, they tried to accept the child’s disease by adjusting their minds for minimizing stress. ^27^ It seems that in different cultures families adjust themselves to their child’s disease by various ways based on their beliefs, so they gradually adjust their lifestyle.

In our study, optimism and hope for the child’s recovery and life were among the coping strategies which were effective in continuation of caregiving and treatment of the child. Optimism can influence physical and psychological well-being by improving adaptive behaviors and a healthy lifestyle. ^32^ In other studies, the families employed optimism and lack of concern for the future as a strategy for coping with the child’s heart disease. ^16^ In a study, some mothers lost hope, and it was hard for them to stay positive in the face of their child’s critical condition, which is inconsistent with our study. ^33^ Optimism about treatment and future led the families to gain hope and create a happy and calm home environment; also, hope played an important role in coping with difficult conditions; ^34^ moreover, the progress in child’s recovery was a positive experience for the family. ^24^ In addition, family members’ sympathy and hopeful sentences had a great effect on the parents. ^35^ These findings highlight the importance of the efforts of the nurses and other health professionals in helping families towards adopting positive thinking and optimistic attitude, and encouraging them to strengthen their hope in the caregiving process.

In our study, the participants tried to tolerate difficult conditions of caregiving, control themselves, and behave patiently with the child and family members. These actions may result from the sense of commitment and responsibility towards taking care of the child and other family members to maintain a calm atmosphere in the family. Creating a peaceful environment may have positive effects on the physical and psychological health of the family members. In a study, self-restraint was one of the dimensions of commitment to care and was considered as a strategy to cope with caregiving situation which was related to patience and tolerance. ^36^ In a similar study, mothers attempted to resist hard feelings despite their worries about the future of their child’s disease. ^31^ Moreover, in a study, self-control had a positive effect on mental health which improved the ability to manage behavioral responses to stressful situations. On the other hand, individuals with low self-control tended to use unhealthy coping strategies. ^37^ Culture may influence the perceived difficulty of situations, so it may be effective in coping with the situation. ^30^

In the present study, families sought emotional and social support from their husband/wife, family, physicians, and relatives to get calm and satisfy their psychological needs. In other studies, in addition to the above-mentioned sources of support, the families received support from their peers, online support groups, other sources in the hospital, workplace, and the community. ^13 - 15 , 24 , 38^ Since culture can influence the perceived social support, ^30^ sources of support can be various due to diversities in the health systems and available sources in different societies and cultures. According to a study, European Americans seek for social support more frequently than Asians and Asian Americans. ^39^ In a study in Iran, high perceived social support in patients was related to marital status and the importance of religiosity in Iranian Muslim culture. ^40^ It seems that in Iranian culture, support is more provided by family members and relatives, and other sources of social and professional support play fewer roles, which can be due to the lack of awareness and access to these sources. Providing social and professional support may be very important in the families’ ability to meet their needs in child caregiving.

In this study, the participants used different coping strategies in stressful situations of caregiving. Some participants used maladaptive (negative) coping strategies like crying and aggression to release their emotions. In a study, parents of children with CHD used maladaptive coping strategies, such as denying, worrying, crying, and adopting aggressive behavior with medical staff, ^16^ which were consistent with our study. Furthermore, in our study, some participants used other coping strategies such as watching TV, reading books, spending time out of home as distracting techniques for releasing emotions. In a study, parents used distracting techniques (i.e. going out for dinner or playing card) to reduce worrying thoughts while their child was staying in cardiac intensive care unit. ^33^ In a study, mothers of children with CHD felt more relieved by releasing emotions when they met other families in the same situation. ^8^ Coping strategies which reduce emotional distress through venting emotions and engagement in distracting activities are referred to as emotion-focused coping. Cultural values are predictors of emotion-focused/avoidant coping which are much more common in non-western cultures. ^30^ Nurses and other health care professionals may help the families by assessing the situations and teaching them adaptive coping strategies. 

In the present study, the participants used some strategies to manage their family, and their own psychological needs, including the consuming sedative medications, consulting with psychiatrists, paying attention to other family members, and socializing with friends. In similar studies, the families tried to cope with their conditions using different strategies, including trying to increase their own physical and mental health, devoting time to regain their energy, paying attention to their other children, trying to improve their relationships, using proactive problem solving, and being strong and proactive through work. ^8 , 16^ Coping strategies in which people try to solve their challenges by modifying or managing the problem are referred to as problem-oriented coping. ^30^ In a study, some parents experienced feeling isolated and difficulty sleeping in coping with challenges that had led to depression and suicidal thoughts; this is inconsistent with our study. ^16^ However, in our study, parents faced challenges in devoting time and paying attention to themselves and other family members, as well as managing their routine activities. 

The present study revealed the coping strategies used by families in the face of caregiving stress. These results can help the nurses and health care professionals to design educational, supportive, and psychological interventions, along with coping strategies used by families, for making families’ efforts more effective in coping with caregiving stress and maintaining their well-being. Therefore, it may facilitate the caregiving process.

The strength of our study was the exploration of the experiences of all family members including parents, siblings and grandmothers, whereas other studies had focused on mothers’ or parents’ experiences. The limitation of this study was that the results may not be generalized because the participants were in a particular geographical region, and nature of the qualitative research method limits the generalizability. Another limitation could be using a telephone interview with a child’s sister because the participant may not have fully shared her experiences via telephone, and it could be different from the other interviews.

## CONCLUSION

The present study showed that the families of children with CHD used a variety of coping strategies including spirituality, acceptance, optimism, patience, and managing psychological needs based on their beliefs, attitudes, abilities, and available resources in the face of caregiving stress. It is important that nurses and other health care providers be aware of it. Accordingly, they can develop appropriate interventions to help the families cope effectively with stressful situations, and the challenges they face in their child caregiving. Moreover, they may provide the families with appropriate educational programs to strengthen the families’ ability and management skills regarding coping with caregiving stress and challenges based on the needs and priorities of families. It is suggested that effective interventions should include implementation of supportive programs, psychological counseling, family-centered empowerment programs, introduction of social support services, and regular interaction with healthcare providers (i.e. physicians, nurses, and psychiatrists). The effectiveness of the above-mentioned interventions on coping with caregiving stress in the families is recommended to be examined in future studies.
